# Employment status and self-rated health in people with multiple sclerosis in Sweden

**DOI:** 10.1186/s12883-026-04809-5

**Published:** 2026-03-11

**Authors:** Alejandra Machado, Jessica Dervish, Emilie Friberg

**Affiliations:** Division of Insurance Medicine, Department of Clinical Neuroscience, Karolinska Institutet, Stockholm, SE-171 77 Sweden

**Keywords:** Employment, Self-rated health, Mental health, Sickness absence, Disability pension

## Abstract

**Introduction:**

In Sweden, many people with multiple sclerosis (PwMS) remain employed due to flexible work and social insurance support. However, as MS progresses, work income declines while reliance on sickness absence (SA) and disability pension (DP) increases. Self-rated health, a holistic measure of physical and mental well-being, is informative in the work context. This study aims to explore factors associated with combined categorical indicators that integrate employment status and self-rated health and examines factors associated with different employment-health states.

**Methods:**

This population-based study included survey responses from 4329 PwMS linked to national register data. We categorized participants into four employment–health groups based on self-rated health (high vs. low EQ-VAS) and employment status (working vs. receiving SA/DP). Groups comparisons were conducted to describe differences in sociodemographic, clinical, mental health, and work-related characteristics. Multinomial logistic regression was then used to examine factors associated with group membership.

**Results:**

Women, older individuals, and those with progressive MS or more visible symptoms were more likely to be receiving SA/DP. Depressive symptoms were more common among participants reporting low self-rated health, regardless of employment status, while disability, fatigue, and reduced work ability were associated with poorer health and reduced labor market participation. Fatigue emerged as a prevalent symptom across all groups, including those still working. Participants working despite low health reported insufficient or absent workplace adjustments, suggesting unmet support needs. Career and educational decisions were also influenced by MS, with many participants reducing working hours or avoiding job changes.

**Conclusions:**

Employment status among PwMS did not perfectly reflect individual’s overall well-being, particularly regarding mental health and fatigue. Nevertheless, clear group-level differences between those who were employed and those who were receiving part-time SA/DP underscore the importance of early, tailored interventions.

## Introduction

Multiple sclerosis (MS) can significantly affect an individual’s working life, where people with MS (PwMS) tend to have lower employment rates than those without MS, and participation in work often declines over time [1–3]. Despite this trend, Sweden has a relatively high proportion of working-aged PwMS in paid employment, [4] often facilitated by part-time work and flexible arrangements supported by the national social insurance system [1, 4, 5]. However, as the disease progresses, income from paid work often decreases, while reliance on sickness absence (SA) and disability pension (DP) increases [2, 3].

Multiple factors can influence employment outcomes among PwMS, including disease-related factors physical limitations, symptom burden, and disability level, [6] as well as mental health challenges such as fatigue, depression, anxiety, and cognitive difficulties. These ‘invisible symptoms’, can be highly disruptive to sustained work performance and strongly influence individuals’ perceptions of their work ability [7–9]. Moreover, coping strategies and psychological distress can act as barriers to remaining in work [10, 11]. Previous studies have shown a strong association between health status and employment outcomes in PwMS, [12, 13] including evidence from Nordic countries [14]. Furthermore, Sweden’s 2008 law changes increased employers’ duty to adapt work and support employees to prevent early labor‑market exit [15]. Certainly, workplace conditions and adjustments play important roles as well. Flexible hours, opportunities for remote work, and ergonomic support have all been shown to help PwMS continue working [16]. Supportive managers and inclusive work environments further increase the likelihood of job retention, especially when adjustments are offered at the first sign of difficulty or following the employee’s request, rather than when disability becomes evident [17]. Therefore, it is important to consider both physical and mental health challenges when assessing employment outcomes.

In this context, overall self-rated health, as measured by the EuroQol-visual analogue scale (EQ-VAS), is a critical yet underexplored factor in employment status among PwMS. Contrary to other narrowly defined health indicators, EQ-VAS captures physical, mental and emotional well-being, offering a holistic view of how individual’s perceive their health [18]. Indeed, differences between EQ-VAS and other measures suggest it reflects a broader health construct, potentially providing a summary more aligned with the patient’s perspective [19].

While being employed is often considered an indicator of wellbeing, it does not necessarily reflect the underlying health status of individuals [20]. Many PwMS continue working despite significant mental and cognitive challenges [7]. To better understand these patterns, we consider sociodemographic factors (e.g., age, sex, education, living area) and mental health indicators (e.g., depression, anxiety, fatigue) as potential correlates of differences across the combined employment–health groups. Accordingly, this study aims to characterize distinct employment-health groups among working-aged PwMS and examine sociodemographic, clinical, cognitive, mental health, and work-related factors associated with membership in these groups.

## Methods

### Study design and setting

An observational study was conducted based on a cross-sectional survey of PwMS in Sweden. Data collection was accomplished from May to September 2021 and linked to individual-level register data by Statistics Sweden.

### Study sample

All individuals living in Sweden between the ages of 20–50 years and included in the Swedish Multiple Sclerosis Registry (SMSreg) were invited to participate in a web-based survey administered by Statistics Sweden. Of those invited, 4412 (52%) responded to the survey. The present study includes 4329 participants who provided information related to current employment status (60 missing) and self-report health (23 missing).

### Data sources

The survey data collected was linked to the following national register data:

The Longitudinal Integrated Database for Health Insurance and Labour Market Studies (LISA), held by Statistics Sweden, was used to obtain socio-demographic variables as of 31 December 2019 (sex, educational level, country of birth, marital status, living with children at home, and type of living area). Age was calculated as the difference between the day of birth and the date of survey response.

The Swedish MS register (available up to 2021), administered by Region Stockholm, which includes clinical information such as time since diagnosis, type of MS (relapsing-remitting, secondary-progressive, primary-progressive, missing); MS disability, assessed with the most recently available Expanded Disability Status Scale score (EDSS) and occurring within three years of the survey response (*n* = 442 set to missing), and participants with secondary-progressive or primary-progressive MS with an EDSS score of 0 were set to missing (*n* = 13) [21]. The scores were then categorized (as mild (EDSS = 0–2.5), moderate (3–5.5), and severe (6–9.5) or missing). Cognitive processing speed scores assessed with the symbol digit modalities test (SDMT), [22] were only used if measured within three years of the survey response (*n* = 523 set to missing).

### Survey measures

The web-based survey was developed within our research group in close collaboration with clinicians and patient representatives and has been published elsewhere [23]. Several closed-ended questions were selected from the survey as measures of overall health outcomes such as mobility, self-care, usual activities, pain/discomfort and anxiety/depression (measured by EQ-5D-3L) [24, 25], and more defined fatigue, depression, and anxiety subscales (Neuro-QoL), [26] which were later included as explanatory variables in the regression models.

Further closed-ended questions on work-related outcomes, such as impact of MS on work, career choices and perceived work ability were explored through the following questions: ‘*How important is work in your life?*’; ‘*What is your current work ability compared with when it was at its best?*’; ‘*Do you have any adjustments or support that helps you with your work?*’; ‘*Apart from MS*,* do you have another long-lasting disease*,* diagnosis*,* or disability?*’; ‘*Have any of these affected your choice of profession or work situation?*’; and ‘*Has your MS diagnosis contributed to you doing any of the following?*’(Please see Table 2, as well as Fig. 2 for the categorization of possible answers).

In addition, an open-ended question was included that related to reported MS restrictions. Responses from this open-ended question, “*Which MS symptom do you experience as the most limiting*?” informed on the most limiting symptom and were grouped into 3 categories following the European and American MS guidelines for symptom management, as reported elsewhere [21].

### Creation of employment-self-rated health groups

Employment status was defined based on participants’ self-reported current employment situation and were categorized into five employment status groups: (1) Working and no reported sickness absence (SA) or disability pension (DP); (2) Other situations (e.g., studying, on parental leave, unemployed or job seeking) but no SA or DP reported; (3) Working combined with part-time SA; (4) Working combined with part-time DP; (5) Not working (either full SA or DP). For clarity, the working definition included individuals who were either employed or self-employed.

Self-rated health was measured with EQ-VAS (a survey item); a scale ranging from 0 (worst imaginable health) to 100 (best imaginable health), where respondents rate their overall health status. EQ-VAS scores have shown consistency with the severity of health states from the EQ-5D-3L [27]. Based on the study sample’s median EQ-VAS (*M* = 75), a high (≥ 75) or low (< 75) health status was defined, in line with common Health Related Quality of Life (HRQoL), [28] with the study mean falling below that report for the general Swedish population (median = 80) [29]. Furthermore, to capture the heterogeneity of experiences among people with MS, we created a combined employment–health indicator, grouping participants by employment status (working or other situations without SA/DP, and partially working or not working due to SA/DP) and self-rated health (high vs. low). This allowed us to descriptively characterize distinct groups and examine which socio-demographic, clinical, and work-related factors were associated with membership in these groups. The following groups were created:

1. Working & high health
2. Working & low health
3. SA/DP & high health
4. SA/DP & low health

### Insurance system in Sweden

People in Sweden aged 16 and older who earn income from work or receive unemployment benefits are covered by the national sickness absence (SA) insurance if illness or injury reduces their work capacity. Employers provide sick pay for the first 14 days, after which the Social Insurance Agency pays SA benefits; unemployed individuals receive benefits from the Agency starting on the first day. A medical certificate is required after seven days of illness. Residents aged 19–64 who experience long-term or permanent work incapacity may qualify for disability pension (DP), and both SA and DP benefits can be granted at 25%, 50%, 75%, or 100% of normal working hours [30].

### Statistical analysis

The study sample was described with frequencies and proportions for the categorical covariates and median values with an interquartile range (IQR) for continuous covariates. Group proportions were assessed across all four groups, with Chi-square tests of independence, while median comparisons were calculated with Kruskal-Wallis’s test. Additionally, targeted comparisons were conducted between the two intermediate groups - those working with low health and those on SA/DP with high health - to explore finer distinctions. All pairwise comparisons were corrected using the Benjamini-Hochberg procedure to control the false discovery rate.

Multinomial logistic regression was used to estimate odds ratios (OR) and 95% confidence intervals (CI) for associations between group membership and previously explored factors, using a stepwise approach that retained only variables with statistical significance and supported by literature. The outcome categories represented distinct employment–health states. Although multinomial logistic regression relies on the independence of irrelevant alternatives (IIA) assumption, the categories reflect distinct states rather than substitutable choices, and the analyses were therefore used to descriptively characterize group differences rather than infer causality. Sensitivity analyses using continuous EQ-VAS and additional health-related covariates were conducted to assess the robustness of the findings as well as multivariate models without work-related factors that could bias sample selection. All statistical analyses were performed using SPSS v29, and a *p*-value of < 0.05 was considered statistically significant.

## Results

### Baseline characteristics

The working groups presented similar demographic characteristics, regardless of whether participants reported high or low health status on the EQ-VAS (Table 1). Specifically, about two-thirds of the participants were women, with the largest group represented between 40 and 49 years (~ 48%) and resident in cities (~ 47%). In contrast, the SA/DP groups had higher percentages of women (76–83%), with 83% in the high health group and 76% in the low health group. Additionally, a greater proportion were older (61% and 54% in the high and low health groups, respectively) and lived mainly in towns or suburbs (41%). A clear stratification in educational level was evident, with lower attainment in the SA/DP groups. The proportions of university-level education were 60% in the working and high health group, 55% in the working and low health group, 42% in the SA/DP and high health group, and 38% in the SA/DP and low health group (Table 1).

**Table 1:** Demographic and clinical characteristics across four groups and intermediate employment-health comparisons

	Working & high health (group 1)		Working & low health (group 2)	SA/DP & high health (group 3)	SA/DP & low health (group 4)	Overall *p*-value (4 groups)	Group 2 vs. 3 *p*-value
	(*n* = 2248)		(*n* = 1049)	(*n* = 238)	(*n* = 794)		
*DEMOGRAPHIC DATA*							
Sex, *n* (%)						**< 0.001**	**< 0.001**
Women	1551 (69.0)		733 (69.9)	197 (82.8)	606 (76.3)		
Men	697 (31.0)		316 (30.1)	41 (17.2)	188 (23.7)		
Age group, *n *(%)						**< 0.001**	**< 0.001**
20–29 years	213 (9.5)		106 (10.1)	16 (6.7)	50 (6.3)		
30–39 years	773 (34.4)		364 (34.7)	45 (18.9)	208 (26.2)		
40–49 years	1106 (49.2)		501 (47.8)	145 (60.9)	430 (54.2)		
50 + years	156 (6.9)		78 (7.4)	32 (13.4)	106 (13.4)		
Median [IQR]	41.0 [35–46]		41.0 [34–46]	44.5 [39–48]	43.0 [38–48]		
Educational Level, *n *(%)						**< 0.001**	**< 0.001**
Non-university	911 (40.5)		471 (44.9)	138 (58.0)	493 (62.1)		
University/college	1337 (59.5)		578 (55.1)	100 (42.0)	301 (37.9)		
Born in Sweden, *n *(%)						0.388	0.514
No	250 (11.1)		135 (12.9)	27 (11.3)	102 (12.8)		
Yes	1998 (88.9)		914 (87.1)	211 (88.7)	692 (87.2)		
Children < 18 at home, *n *(%)						**0.003**	0.650
No	929 (41.3)		477 (45.5)	112 (47.1)	392 (49.4)		
Yes	1318 (58.7)		569 (54.2)	127 (52.9)	401 (50.5)		
Married/cohabitant, *n *(%)						**0.004**	0.407
No	1207 (53.7)		621 (59.2)	134 (56.3)	470 (59.2)		
Yes	1041 (46.3)		428 (40.8)	104 (43.7)	324 (40.8)		
Type of living area, *n* (%)						**< 0.001**	**0.010**
City	1041 (46.3)		502 (47.9)	90 (37.8)	295 (37.2)		
Town/suburb	865 (38.5)		387 (36.9)	97 (40.8)	325 (40.9)		
Rural area	342 (15.2)		160 (15.3)	51 (21.4)	174 (21.9)		
*CLINICAL DATA*							
EDSS category, *n* (%)						**< 0.001**	**< 0.001**
Mild [0–2,5]	1709 (76.0)		677 (64.5)	130 (54.6)	286 (36.0)		
Moderate [3–5,5]	114 (5.1)		144 (13.7)	47 (19.7)	210 (26.4)		
Severe [6–9,5]	< 10 (0.2)		29 (2.8)	17 (7.1)	119 (15.0)		
Missing*	421 (18.7)		199 (19.0)	44 (18.5)	179 (22.5)		
Disease duration (years)						**< 0.001**	**< 0.001**
Median [IQR]	7.0 [4–13]		7.00 [3–12]	12 [7–17]	10.0 [5–16]		
Type of MS, *n* (%)						**< 0.001**	**0.001**
RRMS	2179 (96.9)		968 (92.3)	204 (85.7)	286 (36.0)		
PPMS	17 (0.8)		26 (2.5)	< 10 (2.5)	210 (26.4)		
SPMS	31 (1.4)		50 (4.8)	26 (10.9)	119 (15.0)		
Missing*	21 (0.9)		< 10 (0.5)	< 10 (0.8)	179 (22.5)		
SDMT						**< 0.001**	**< 0.001**
Median [IQR]	58 [51–66]		56 [50–63]	52 [45–59]	51[42–60]		
Depression						**< 0.001**	**< 0.001**
Median [IQR]	45.3 [45.3–50.6]		52.1 [46.8–57.4]	48.9 [43.1–53.6]	55.1 [50.6–60.6]		
Anxiety						**< 0.001**	**< 0.001**
Median [IQR]	45.9 [45.9–51.4]		53.3 [46.2–58.4]	48.4 [36.4–54.2]	55.9 [49.5–60.9]		
Fatigue						**< 0.001**	**< 0.001**
Median [IQR]	44.7 [44.7–51.3]		54.4 [49.3–59.9]	52.3 [46.5–58.8]	59.9 [56.5–63.5-5]		
The most limiting symptom, *n* (%)						**< 0.001**	0.199
No symptoms	466 (20.7)		45 (4.3)	10 (4.2)	< 10 (0.6)		
Visible symptoms	362 (16.1)		198 (18.9)	35 (14.7)	162 (20.4)		
Invisible symptoms	1191 (53.0)		742 (70.7)	186 (78.2)	598 (75.3)		
Missing	229 (10.2)		64 (6.1)	< 10 (2.9)	29 (3.7)		

*Missing data from registers not included in the comparisons

This table presents demographic and clinical characteristics for the total population, comparisons among all four groups, and intermediate comparisons between groups 2 and 3, categorized by employment status and self-rated health

*Abbreviations*: *IQR* Interquartile Range, *SDMT* Symbol Digit Modalities Test, *EDSS* Expanded Disability Status Scale, *RRMS* Relapsing-Remitting Multiple Sclerosis, *SPMS* Secondary Progressive Multiple Sclerosis, *PPMS* Primary Progressive Multiple Sclerosis

Note_(1)_: *P*-values in bold indicate statistically significant results

Note_(2)_: Sample size can vary according to response rate of each question

Note_(3)_: To ensure anonymity, frequencies below ten are reported as < 10

Note_(4)_: Scores on all Neuro-QoL subscales (depression, anxiety, and fatigue), are expressed using a standardized T-score metric

Regarding clinical characteristics, obtained from the MS register, participants in the SA/DP groups had longer disease duration, higher percentages of moderate and severe EDSS, and more progressive types of MS (primary and secondary progressive MS, respectively) (Table 1). Compared to the rest of groups, the SA/DP and low health group had higher MS disability (EDSS) and a higher proportion of individuals with PPMS (26%), and SPMS (15%). Finally, lower scores of cognitive processing speed (SDMT) were observed among the SA/DP groups, in comparison to the working groups (*p*<.001). Specific aspects of mental health were assessed using the Neuro-QoL, which revealed higher levels of depression, anxiety, and fatigue among participants in both SA/DP groups, regardless of health status, as well as among those working but reporting low health (*p*<.001). Notably, fatigue scores were consistently higher than those for depression or anxiety across all three groups, suggesting that fatigue was a more prominent issue among the participants (Table 1). 

Among the intermediate groups, demographic differences were observed only in sex, age, educational level and type of living area (Table 1, comparison between group 2 vs. 3). Compared to the working group with low health, the SA/DP group with high health included a higher proportion of women, older individuals, lower educational attainment, and more residents of towns or rural areas rather than cities (*p*<.010). Clinically, the SA/DP group with high health had a longer average disease duration, higher levels of MS-related disability, a greater proportion of progressive MS types, and lower cognitive processing speed (SDMT) compared to the working group with low health (*p*<.001). Interestingly, the working group with low health reported worse scores for depression, anxiety, and fatigue compared to the SA/DP and high health group (*p*<.001). No significant differences were found between the two intermediate groups regarding the most limiting symptom reported (Table 1).

### Other specific survey self-rated health measures

**Fig. 1:**
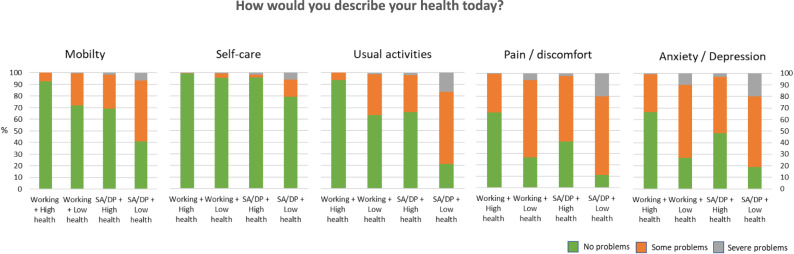
Distribution of EQ‑5D‑3L responses (Mobility, Self‑care, Usual activities, Pain/Discomfort, and Anxiety/Depression) across four groups defined by employment status and self‑rated health. Bars represent the proportion of participants reporting no problems (green), some problems (orange), or severe problems (grey) within each domain. This figure provides a descriptive overview of domain‑specific health differences across the combined employment–health groups and is intended to illustrate patterns contributing to overall self‑rated health, rather than to imply independent or causal associations

To explore the components underlying low or high overall self-rated health, we descriptively examined responses across the EQ-5D-3L dimensions (Fig. 1). Although EQ-VAS and EQ-5D-3L are conceptually related, EQ-5D-3L dimensions were used to illustrate differences in domain-specific self-reported health across the combined employment–health groups, rather than to re-examine established associations between these measures.

Across the four groups, the proportion of participants reporting moderate to severe problems in mobility, self-care, and usual activities increased progressively. This pattern reflects systematic differences in domain-specific health profiles across employment–health states, with fewer reported problems among those working with high health and a greater burden of problems among those receiving SA/DP with low health.

When focusing on the two intermediate groups, no statistically significant differences were observed in the mobility, self-care or usual activities domain (*p* >.05). However, participants who were working despite low self-rated health reported significantly higher proportions of moderate and severe pain/discomfort domain (χ^2^ = 19.085; *p* <.001) and anxiety/depression domain (χ^2^ = 44.019; *p* <.001) compared with participants receiving SA/DP despite high self-rated health (Fig. 1).

### Survey measures related to work

**Fig. 2:**
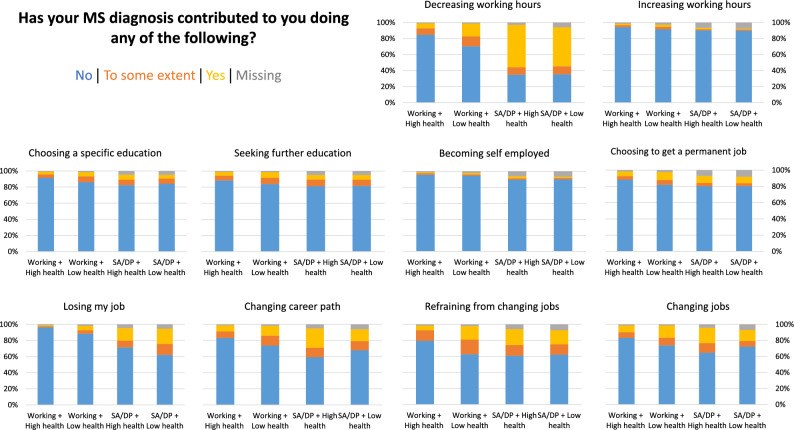
Survey response distribution on MS impact by employment status and self-rated health groups. Impact of MS on career and educational choices, assessed by asking how the diagnosis influenced actions such as decreasing or increasing working hours, choosing or seeking education, becoming self-employed, obtaining a permanent job, losing a job, changing career path, refraining from changing jobs, or changing jobs. Bars illustrate response rates (percentages) for each question: ‘No’ (blue), ‘To some extent’ (orange), ‘Yes’ (yellow), and missing (grey)

Since employment status by itself cannot adequately represent the complexities of working with MS, we explored other work-related characteristics such as type of occupation, workplace support, work ability, and impact of MS in work (Table 2; Fig. 2). Office work was the most common type of occupation reported by all participants, especially those in the working groups (75%). Furthermore, adjustments or support at work were more prevalent among the SA/DP groups. However, a considerable percentage in the working group with low-health reported lacking adjustments (19%) or that these were insufficient (8%). The workplace support differed significantly from those reported by the SA/DP group with high-health, who - despite having adjustments to a larger extent (26%) – were less likely to report needing additional adjustments (5%) or that these were not enough (4%) (Table 2). Notably, the perceived importance of work in life was rated relatively high across all groups, although slightly lower among the SA/DP participants (Table 2).

**Table 2:** Occupation and work-related characteristics across four groups and intermediate comparison (Groups 2 vs. 3)

	Working & high health	Working & low health	SA/DP & high health	SA/DP & low health	Overall *p*-value	Group 2 vs. 3 *p*-value
	Group 1	Group 2	Group 3	Group 4	(all 4 groups)	
	(*n* = 2248)	(*n* = 1049)	(*n* = 238)	(*n* = 794)		
Occupation^+^, *n* (%)					**< 0.001**	**0.001**
Manager	314 (14.0)	135 (12.9)	< 10 (2.5)	22 (2.8)		
Office	1693 (75.3)	782 (74.5)	140 (58.8)	370 (46.6)		
Manual	160 (7.1)	79 (7.5)	< 10 (3.8)	38 (4.8)		
N/A*	81 (3.6)	53 (5.1)	83 (34.9)	364 (45.8)		
Do you have any adjustments/support that helps you with your work?^+^ *n* (%)					**< 0.001**	0.733
Yes	159 (7.1)	156 (14.9)	61 (25.6)	164 (20.7)		
Yes, but not enough	45 (2)	84 (8)	< 10 (3.8)	64 (8.1)		
No, but I do need	123 (5.5)	194 (18.5)	12 (5)	74 (9.3)		
No, not needed	1845 (82.1)	569 (54.2)	71 (29.8)	99 (12.5)		
Missing*	76 (3.4)	46 (4.4)	85 (35.7)	393 (49.5)		
What is your current work ability compared with when it was at its best? (Cannot work at all 1–11 My work ability is currently at its best)					**< 0.001**	**0.001**
Median [IQR]	10 [3–11]	8.0 [1–11]	7.0 [1–11]	4.0 [1–11]		
Missing	0 (0)	0 (0)	2 (0.8)	10 (0.3)		
How important is work in your life? (one of the less important things 1–5 one of the most important things)					**< 0.001**	**< 0.001**
Median [IQR]	4 [4–5]	4 [3–5]	4 [3–4]	3 [2–4]		
Missing	2 (0.1)	0 (0)	2 (0.8)	19 (2.4)		
Apart from MS, do you have another long-lasting disease, diagnosis, or disability?					**< 0.001**	0.859
No	1727 (76.8)	680 (64.8)	156 (65.5)	401 (50.5)		
Yes**	508 (22.6)	354 (33.7)	79 (33.2)	378 (47.6)		
Missing*	13 (0.6)	15 (1.4)	< 10 (1.3)	15 (1.9)		
If yes**, have any of these affected your choice of profession or work situation?					**< 0.001**	0.733
No	399 (17.7)	227 (21.6)	53 (22.3)	180 (22.7)		
yes	104 (4.6)	124 (11.8)	26 (10.9)	190 (23.9)		
Missing*	1745 (77.6)	698 (66.5)	159 (66.8)	424 (53.4)		

^+^To note, the work‑adjustment question was skipped by participants who reported being “not working” (e.g., full‑DP), whereas all other work‑related items were completed by the full sample. Hence, group comparison is applied to individuals who maintain some connection to work (e.g. part-time SA or DP)

* Not applicable (N/A) or missing values (mostly related to those who responded “not working”) were not included in the group comparisons

**All yes answered include previous SA, ongoing DP or ongoing sick leave shorter than 14 days, and therefore not qualifying for sickness compensation

This table presents occupation and other work-related characteristics for the total population, overall comparisons among all four groups, and intermediate comparisons between Groups 2 and 3, categorized by employment status and self-rated health groups. *P*-values in bold indicate statistically significant results

Sample size can vary according to response rate of each question. To ensure anonymity, frequencies below ten are reported as <10

Importantly, the work‑adjustment question was skipped by participants who indicated they were “not working” when asked about their occupation (e.g., full‑DP), while all other work‑related items were completed by the full sample. All participants were asked to rate their current work ability compared to when it was ‘at its best.’ Once more, systematic differences were observed across the four groups, with the highest scores reported by those working and in good health, and the lowest by those on SA/DP with poor health (Table 2). Comparisons among the intermediate groups mirrored those from the overall (four-group) comparison. Specifically, the working group with lower health had higher proportion of people with higher perceived work ability compared to those in SA/DP group with high health (Table 2).

Regarding impact of MS on work and career choices, only a few participants reported increasing their working hours or becoming self-employed (Fig. 2). In contrast, around 60% of individuals in both SA/DP groups indicated a reduction in working hours. MS also influenced decisions to avoid or pursue changing jobs, with a slightly greater impact observed in the SA/DP with high health group compared to the low health group. Job loss was observed in most groups, to varying degrees, affecting the working with low health group and both SA/DP groups. Choosing permanent employment, pursuing further education, or selecting specific fields of study were similarly reported across all groups (ranging from 4% to 15%) (Fig. 2).

Comorbidity was explored among participants, revealing that nearly half (48%) of PwMS in the SA/DP groups, regardless of self-rated health, reported having other long-term diseases, diagnoses, or conditions unrelated to MS. In comparison, only 23% of the working and high-health group reported having additional conditions (Table 2). The working and low-health group and the SA/DP and high-health group had similar response rates (approximately 33%). Among those who reported other diagnoses or conditions besides MS, many indicated that these had impacted on their ability to work (24%) and had led to previous (12%) or ongoing sickness absence (13%), or ongoing disability pension (14%) (Table 2).

### Associations with employment–health group membership

Based on the descriptive patterns observed across the four employment–health groups, we operationalized covariates in the multivariable model to capture factors likely associated with group membership. Sociodemographic variables included sex, age, education, and type of living area; clinical variables comprised disease duration, MS type, and EDSS; cognitive function was represented by SDMT scores; mental health by Neuro-QoL measures of depression, anxiety, and fatigue; and work-related factors included occupation type, workplace support, perceived work ability, and impact of MS in work. Variables were first tested independently, and those significant in univariate analyses or supported by prior literature were retained in the multinomial logistic regression to identify independent associations with group membership.

Results from the final adjusted multivariate model are presented in Table 3. Men were less likely to be in the SA/DP groups compared to women, this was irrespective of high (OR=0.317, 95% CI=0.173–0.583) or low health (OR=0.444, 95% CI=0.277–0.712). Younger PwMS were less likely to be found in the SA/DP groups. Furthermore, individuals with mild disability (EDSS = 0–2.5) were less frequently represented in the low-health working group or both SA/DP groups, while severe disability was more common in the low-health SA/DP group (OR = 3.743, 95% CI = 2.315–6.051). Depression was more prevalent among PwMS in the low health groups, regardless of employment status - working (OR = 1.052, 95% CI = 1.034–1.070) or on SA/DP (OR = 1.051, 95% CI = 1.021–1.081). Fatigue had a high association with all groups, though slightly less so among the SA/DP with high-health group than the working and low-health or SA/DP and low-health. Visible symptoms were also more prominent among participants in the SA/DP groups. Furthermore, participants on SA/DP had higher likelihood of reporting having more workplace adjustments (*p* <.001). The SA/DP and low health had higher likelihood of poorer working ability (OR=0.333, 95% CI=0.296–0.375) compared to the reference group - those working and high health. As expected, individuals in the working group and low-health were less likely to have SA or DP due to other significant comorbidities (Table 3). As the inclusion of work adjustment in the multivariate model could introduce selection bias by excluding participants who were not working, we re‑estimated the model without this variable, and the findings remained consistent (data not shown).

**Table 3:** Adjusted multinomial logistic regressions with ‘working and high health’ as reference (*n* = 2,748)

	Working & low health			SA/DP & high health			SA/DP & low health		
	AOR	95% CI	*p*-value	AOR	95% CI	*p*-value	AOR	95% CI	*p*-value
Sex									
men	1.127	(0.888 − 1.430)	0.325	0.317	(0.173–0.583)	**< 0.001**	0.444	(0.277–0.712)	**< 0.001**
Age (cont.)	1.008	(0.993–1.023)	0.305	1.086	(1.050–1.124)	**< 0.001**	1.060	(1.030–1.091)	**< 0.001**
Educational level									
non-university	0.859	(0.691–1.069)	0.173	1.450	(0.959–2.192)	0.078	1.145	(0.789–1.661)	0.476
EDSS categories									
moderate - severe	1.833	(1.298–2.590)	**< 0.001**	2.104	(1.180-0.3751)	**0.012**	3.743	(2.315–6.051)	**< 0.001**
Type of MS									
progressive (PP & SP)	2.153	(1.195–3.879)	**0.011**	3.006	(1.306–6.915)	**0.010**	1.839	(0.852–3.973)	0.121
Depression	1.052	(1.034–1.070)	**< 0.001**	0.983	(0.952–1.015)	0.303	1.051	(1.021–1.081)	**< 0.001**
Fatigue	1.062	(1.042–1.082)	**< 0.001**	1.041	(1.004–1.079)	**0.029**	1.077	(1.041–1.113)	**< 0.001**
Most limiting symptom									
no symptom	0.786	(0.516–1.196)	0.261	0.374	(0.109–1.284)	0.118	0.383	(0.093–1.579)	0.184
visible symptoms	1.179	(0.884–1.572)	0.264	0.261	(0.127–0.538)	**< 0.001**	0.544	(0.316–0.938)	**0.028**
Work ability	0.623	(0.573–0.677)	**< 0.001**	0.494	(0.431–0.565)	**< 0.001**	0.333	(0.296–0.375)	**< 0.001**
Work adaptations*									
Yes	1.126	(0.814–1.558)	0.472	2.650	(1.623–4.325)	**< 0.001**	2.959	(1.842–4.753)	**< 0.001**
Yes, but not enough	1.376	(0.838–2.258)	0.207	0.910	(0.355–2.333)	0.844	1.710	(0.859–3.404)	0.127
No, but needed	1.291	(0.920–1.813)	0.140	0.564	(0.253–1.255)	0.161	0.862	(0.489–1.521)	0.608
SA/DP due to other conditions**									
No	1.281	(1.018–1.612)	**0.034**	1.173	(0.751–1.831)	0.483	1.407	(0.960–2.062)	0.080

*Abbreviations: SA* sickness absence, *DP* disability pension, *AOR* Adjusted odds ratios, *CI* confidence interval, EDSS (Expanded Disability Status Scale), *MS* Multiple sclerosis

Covariates included in the model were selected based on either their independent association (for unadjusted logistic regression results, not shown) or on prior evidence from the literature. References (by order): women, university, mild EDSS, relapse-remitting MS; invisible symptoms; No, not needed; Yes. P-values in bold indicate statistically significant results. Fatigue and depression (NeuroQoL subscales) were modeled as standardized T-scores (mean = 50, SD = 10), whereas other variables were included in their original units; odds ratios thus represent changes in group membership per unit increase in each predictor

* To note, the work‑adjustment question was skipped by participants who reported being “not working” (e.g., full‑DP). Hence, this model applies to a selection of participants that had some connection to work and part-time SA or DP

**Operationalization of the question “Apart from MS, do you have another long-lasting disease, diagnosis, or disability?”

Additional sensitivity analyses were also conducted to assess whether defining employment–health states using the overall EQ-VAS could create circularity with other, more specific health measures. When EQ-VAS was modelled as a continuous variable, it was initially associated with all employment–health groups (*p*<.001). However, after including fatigue, depression, and work ability—variables conceptually and empirically related to EQ-VAS—this association was attenuated and no longer statistically significant (*p*=.297), while these specific health measures remained strongly associated with group membership (*p*<.001). These findings indicate that the observed associations in the main analyses reflect relationships with specific health-related constructs overlapping with global self-rated health, rather than being driven solely by the categorization of EQ-VAS.

## Discussion

This study explores the relationship between combined employment-health groups and sociodemographic, clinical, cognitive, mental health and work-related characteristics among working-age individuals with MS. Participants were categorized into four different groups based on self-assessed health (high or low EQ-VAS) and employment: actively working (employed or self-employed) without sickness absence (SA) or disability pension (DP), or receiving part- or full-time SA/DP benefits. Differences across groups were observed in health outcomes, work ability, and disease severity. Participants receiving SA/DP, particularly those with low self-rated health, exhibited more severe clinical profiles, greater functional limitations, and reduced work capacity. Among individuals who remained employed despite low self-rated health, higher levels of pain, fatigue, and mental burden were reported, reflecting factors that may influence their perception of poorer health.

Our study sample, consisting predominantly of mid-life women, reflects the well-established higher prevalence of MS in this age group, [1, 31] as well as the greater proportion of women receiving SA/DP compared to men [32–34]. Initial differences in educational attainment suggested that education might act as a buffer by providing access to resources, health literacy, and occupational opportunities that can potentially delay or mitigate exit from the labor market [35]. However, once clinical factors were considered, the effect of education was no longer significant, suggesting that disease-related manifestations may play a stronger role in determining work outcomes among PwMS [36]. In this regard, participants receiving part-time or full-time SA/DP exhibited clinical profiles indicative of more advanced MS progression, consistent with previous Swedish studies [1–3, 37]. These patterns were mirrored in self-reported measures, particularly among those on SA/DP with low self-rated health, who had the highest proportion of progressive MS, long disease duration, reported more severe problems and comorbdities [2, 34, 38]. These findings align with prior research showing a decline in functional ability and increased self-reported impact of MS several years post-diagnosis, [37] and with studies linking progressive MS and comorbidities to higher rates of unemployment or inactivity compared to relapsing forms [39, 40].

To further explore what distinguishes individuals who continue working despite poor perceived health from those on SA/DP but reporting good health, we found that the SA/DP group with high self-rated health still reported worse mental health, including higher levels of depression, anxiety, and pain. These results may reflect not only the direct impact of MS but also challenges associated with managing work-life balance with the disease as suggested in previous studies [38, 41–43]. In any case, the increased reporting of moderate to severe problems in functionality and psychological well-being among these groups reflects not only the physical burden of the disease but also the mental health implications, particularly in terms of anxiety and depression. Cognitive performance, measured by SDMT scores remained higher among those working despite low health than among SA/DP with high health, suggesting that cognitive processing speed may be more closely linked to employment status than to overall subjective health perception [8].

Fatigue is a common symptom among PwMS and is linked to significant reductions in quality of life as well as a substantial economic burden [44]. Importantly, fatigue emerged as a critical factor across all groups, including those still in paid work. This aligns with recent evidence from Plow and Gunzler (2022), who demonstrated that fatigue in MS is a multidimensional and prevalent symptom, distinct from but interrelated with cognitive impairment and depression [45]. It is also worth noting that “invisible” symptoms, predominantly fatigue-related, were frequently reported by participants who appeared to be in relatively ‘better’ health. This finding also underscores the limitations of relying exclusively on traditional clinical measures such as EDSS scores or diagnostic categories to assess work capacity, as they may not fully capture the lived experience of these conditions, particularly the “invisible” symptoms like fatigue, pain, and cognitive issues [46]. Further, these findings emphasize the need for an integrated care addressing both physical limitations and mental health challenges of PwMS [47], especially to support their ability to meet work demands [42, 48].

Work ability was influenced by MS-related disability, fatigue, and occupational factors [49]. Our findings suggest that individuals with poorer health who continue working may face gaps in workplace support, including unmet needs for accommodations. Many reported needing adjustments that were either nonexistent or insufficient. A key barrier is the reluctance to request support without disclosing MS, often only done when symptoms become visible or begin to affect job performance [21, 50]. At the same time, the reported need for adjustments also reflects the recognition of many PwMS of their potential to remain in work, which can contribute to improving their quality of life and well-being [23, 51]. However, without timely and appropriate work adjustments (e.g. reduced working hours), continued employment may lead to greater psychological strain and poorer self-rated health, as seen between the working group with low-health compared to the SA/DP group with high-health. In addition, the broader impact of MS on career and educational choices were observed, with rates of pursuing further education, specific study fields, or permanent employment similar across groups, while self-employment remained low [52]. This may reflect that individuals with MS, even early in the disease course, consider educational or career adjustments as part of anticipating future work demands [53], although the relatively young age and mild disease severity of the study population may have limited the extent to which such concerns were expressed.

Altogether, these findings underscore the importance of proactively adapting work demands and offering flexible career support to prevent long-term mental health consequences. They also emphasize the need for employers to adopt more inclusive approaches as a standard practice, rather than waiting for formal disclosure or visible signs of incapacity. Particularly when evidence shows that, with appropriate accommodations, many PwMS can remain employed well beyond the early stages of diagnosis [16, 48].

A key strength of this study is the large sample size and the combination of high-quality Swedish register data with self-reported survey responses, providing a nuanced understanding of the relationship between MS, health status, and employment. These data allow for descriptive characterization of subgroups defined by employment and health status, and identification of sociodemographic and clinical factors associated with these patterns.

Several limitations should be acknowledged. First, the use of a median split to form the four employment-health states may introduce partial mechanical associations with covariates. Accordingly, these comparisons should be interpreted as descriptive, aimed at characterizing subgroup patterns rather than inferring causality. Nevertheless, median splits remain a practical and widely used approach for presenting EQ‑VAS subgroup patterns due to their clear interpretability for clinical and policy audiences [28]. Sensitivity analyses suggested that the observed associations were not solely explained by the median split of EQ-VAS and were compatible with the influence of more specific, conceptually related health measures. Second, the cross-sectional design precludes casual conclusions. Reverse causality and simultaneity remain plausible, as employment status may also influence self‑rated health. While associations cannot be interpreted casually, they can still offer valuable insights for policy by identifying vulnerable groups and highlighting areas suited for future longitudinal work. Third, although the age range of 20–50 years was chosen to reflect the survey’s focus on individuals balancing work and family life, it may limit generalizability to older adults. Fourth, analyses involving variables such as occupation and work‑adjustment were limited to participants who did not report being “not working”; therefore, the multivariate models reflect only this subset of the sample (e.g., excluding those on full‑DP), and these findings should be interpreted with caution. However, additional sensitivity analyses yielded consistent results. In addition, respondents differed from non‑respondents (who were typically younger, male, lower‑income, born outside Sweden, and with shorter disease duration) and only aggregated data were available for non‑respondents. As a result, statistical adjustment for nonresponse was not possible, which may affect generalizability. Further, using demographic characteristics of 2019 may not capture small changes by 2021. However, we consider it unlikely to materially affect our results. Lastly, data collection occurred during the COVID-19 pandemic (May–September 2021), which may have influenced employment or health perceptions [54]. However, a related analysis of the same cohort indicated that many participants reported little change in their work situation, although experiences varied. Thus, pandemic‑related effects were present but are unlikely to have dominated the observed patterns.

## Conclusion

This study shows that employment status among PwMS does not always align with overall well-being, particularly regarding mental health and fatigue. It also highlights the significant impact of disease-related disability, fatigue, and mental burden on employment outcomes among PwMS. Clear differences between those employed and those receiving SA/DP underline the need for early, comprehensive interventions. Supporting individuals through tailored education and workplace strategies, and integrated assessments of both clinical and self-reported symptoms, may help identify factors associated with work ability and inform interventions to maintain employment. Understanding how early support for managing disease-related symptoms, such as fatigue, pain or mental health challenges, contributes to sustained employment, which is vital for enhancing the quality of life and well‑being of PwMS.

## Data Availability

The data in this project is not publicly available in accordance with the General Data Protection Regulation, the Swedish Data Protection Act, the Swedish Ethical Review Act, and the Swedish Public Access to Information and Secrecy Act. Readers may contact Associate Professor Emilie Friberg (emilie.friberg@ki.se) regarding the data.

## Abbreviations

EDSS: Expanded Disability Status Scale
EQ-VAS: EuroQol Visual Analogue Scale
IQR: Interquartile range
MS: Multiple sclerosis
PwMS: People with multiple sclerosis
SMSreg: Swedish Multiple Sclerosis Registry
